# Association of metabolic syndrome with obesity measures, metabolic profiles, and intake of dietary fatty acids in people of Asian Indian origin

**DOI:** 10.4103/0975-3583.70911

**Published:** 2010

**Authors:** Mithun Das, Susil Pal, Ghosh Arnab

**Affiliations:** *Postgraduate Department of Anthropology, Sree Chaitanya College, Habra, West Bengal, India*; 1 *Human Genetic Engineering Research Centre, Kolkata, West Bengal, India*; 2 *Biomedical Research Laboratory, Department of Anthropology, Visva Bharati University, Santiniketan, West Bengal, India*

**Keywords:** Dietary fatty acids, metabolic syndrome, obesity, Asian Indians

## Abstract

**Objective:**

The present community-based cross-sectional study was aimed to examine the association of metabolic syndrome (MS) with obesity measures, metabolic profiles, and intake of dietary fatty acids in Asian Indian population.

**Patients and Methods:**

A total of 350 adult (30 years and above) individuals (184 males and 166 females) inhabiting in and around Kolkata, India participated in this study. MS was defined using the protocol specifically designed for Asian Indian population.

**Results:**

The prevalence of MS in the study was 31.4%. The prevalence was significantly higher (*P* < 0.01) in females (48.2%) as compared to males (16.3%). It was observed that males without MS had significantly higher mean waist circumference (WC *P* < 0.05); waist–hip ratio (WHR; *P* < 0.001); triglyceride (TG; *P* < 0.05); very low density lipoprotein cholesterol (VLDLc; *P* < 0.05) and fasting blood glucose (FBG; *P* < 0.01) as compared to females without MS. Significant differences were also observed for dietary intake of total fatty acids (TFA; *P* < 0.001); saturated fatty acids (SFA; *P* < 0.001) and polyunsaturated fatty acids (PUFA; *P* < 0.001) between individuals with and without MS. However, no significant association was observed in individuals with MS after controlling for age and sex. On the other, WC and body mass index (BMI) had significant correlation with SFA: mono unsaturated fatty acids (MUFA; *P* < 0.01) in individuals without MS even after controlling for age and sex.

**Conclusion:**

It seem reasonable to argue that while dealing with MS in Asian Indians, clinicians should consider obesity measures, metabolic profiles and dietary fatty acids simultaneously.

## INTRODUCTION

The metabolic syndrome (MS) may be defined as the constellation of cardiovascular disease (CVD) risk factors, e.g., dyslipidemia, hypertension, hyperglycemia, etc. Persons with MS are essentially at twice the risk of CVD compared to those without the syndrome. It further raises the risk of type 2 diabetes mellitus (T2DM) by about fivefold. In most countries, about 20–30% of the adult population is predisposed to MS.[1] The MS is not a discrete entity known to be caused by a single factor. Moreover, it shows considerable variation in the components among different individuals. This variation is even greater among different racial and ethnic groups.[2] The MS is not restricted to the adults only, the predisposition of MS however, starts much early in life especially during the adolescence and young age.[3–7]

The prevalence of MS is increasing south Asian countries including India, leading to increased morbidity and mortality due to T2DM and CVD.[8] The increasing incidence of MS among the Asian Indians is a reason for concern since if effective interventions are not applied.[9] Lack of habitual physical activity and certain dietary patterns, including high-saturated fatty acids (SFA) and low vegetable intakes, contribute to weight gain and increase the risk of metabolic disturbances.[10] Saturated fat intake is associated with increased risk of coronary heart disease (CHD); the greatest risk reduction is associated with poly unsaturated fatty acids (PUFA) followed by mono unsaturated fatty acids (MUFA).[11]

In Asian Indians, there existed significant inverse association between central obesity measures and intake of unsaturated fatty acids due to recent shift in dietary habits causing an increase in the prevalence of obesity and dyslipidemia in this region.[12,13] Keeping this view in mind, the present study was undertaken among the Asian Indian population living in the eastern part of India with the following objectives:

- To compare obesity measures, lipids, lipoproteins, plasma glucose, and intake of dietary fatty acids in subjects with and without MS.
- To study the association of dietary fatty acids, their ratios with obesity measures in people with and without MS.

## PATIENTS AND METHODS

### Study population

The present community based cross-sectional study was conducted on adult (aged 30 years and above) Asian Indian men and women from Kolkata (erstwhile Calcutta) and suburbs, West Bengal, India. A total of 350 individuals (184 males and 166 females) participated in the study. Subjects were categorized into two groups: individuals with MS (*n* = 110; male = 30, and female = 80) and without MS (*n* = 240; male = 154, and female = 86). Pregnant women, women on hormone therapy (HT), and individuals with known illness like ischemic heart disease (IHD), T2DM, and hypertension were not incorporated in the study. Before the actual commencement of the study, a public advertisement was circulated regarding the study with the help of local municipal council officials. Individuals were selected randomly after they responded to the local advertisement. The response rate was as high as 85%. The institutional ethics committee of the “Human Genetic Engineering Research Centre” (HGERC) has had approved the study. Written consent was obtained prior to actual commencement of the study.

### Anthropometric measures

Height (nearest 0.1 cm), weight (nearest 0.5 kg), waist (nearest 0.2 cm), and hip circumferences (nearest 0.2 cm) were obtained using standard techniques.[14] The body mass index (BMI, kg/m^2^) and waist–hip ratio (WHR) were subsequently computed.

### Blood pressure

Left arm systolic (SBP) and diastolic (DBP) blood pressure measurements was twice taken using sphygmomanometer and stethoscope and was averaged for analyses. A third measurement was taken only when the difference between the two measurements was ≥5 mmHg. A 5 min relaxation period between measurements was maintained through out the study. SBP and DBP was measured as appearance (phase I) and disappearance (phase V) of Korotkoff sound, respectively.

### Metabolic profiles

A fasting blood sample (7 mL) was drawn from participants for the determination of fasting plasma glucose (FBG), total cholesterol (TC), triglyceride (TG), and high density lipoprotein cholesterol (HDLc). All subjects were maintained an overnight fast of ≥12 h prior to blood collection. Plasma was separated within 2 h of blood collection using a microcentrifuge at 1000 rpm for 20 min in room temperature. Estimation of FBG, TC, TG, and HDLc were carried out using an ERBA Microscan ELISA Reader (Trans Asia Biomedicals Limited, Mumbai, India). Lowdensity lipoprotein cholesterol (LDLc) and very low-density lipoprotein cholesterol (VLDLc) was then calculated using the standard formulas:LDL = TC – (HDL + TG/5) and VLDL = TG/5. All metabolic profiles were estimated in mg/dL (mg %) unit. TC:HDLc ratio was calculated subsequently. The reproducibility of the instruments was checked periodically using control solutions.

### Dietary fatty acids

The dietary intake was recorded using the 24 h recall method for seven consecutive days using a food frequency schedule prepared in local language. The total fatty acids (TFA), saturated fatty acids (SFA), monounsaturated fatty acids (MUFA), and polyunsaturated fatty acids (PUFA) contents of various foods were obtained using the standard guidelines.[15] The standardization to convert foodstuffs in to fatty acids has been mentioned elsewhere.[12,13,16,17]

### Definition of metabolic syndrome

Subjects with any three or more of the following criteria were considered under MS:[18]

- Waist circumference (cm):male > 90; female > 80
- Triglycerides (mmol/L): ≥ 2.25
- HDLc (mmol/L):male < 1.03; female < 1.28
- Blood pressures (mmHg):SBP ≥ 130 and/or DBP ≥ 85
- Fasting blood glucose (FBG) (mmol/L): ≥ 5.56

### Statistical analysis

Descriptive statistics such as mean and standard deviation (SD) of anthropometric, obesity measures, metabolic profiles, and intake of dietary fatty acids were undertaken separately for each group (metabolic vs. non-metabolic) and sex. Person’s partial correlation (adjusted for age and sex) between obesity measures (WC and BMI) and dietary fatty acids was also undertaken. All statistical analyses were performed using SPSS (PC+ version 10). A statistical significance (two-tailed) was set at *P* < 0.05.

## RESULTS

The prevalence of MS in the present population was 31.4%. The prevalence was significantly higher (*P* < 0.01) in females (48.2%) as compare to males (16.3%). The analysis of variance (ANOVA) showed a significantly higher mean WC (*P* < 0.001); WHR (P = 0.001); TC (*P* < 0.001); TG (*P* < 0.001); VLDLc (*P* < 0.001); TC:HDLc (P = 0.05) and FBG in males with MS as compared to females with MS [Table 1].

**Table 1:** Obesity measures, metabolic profiles, and blood pressures variables between males and females with metabolic syndrome (n = 110)

Variables	Male (*n* = 30)	95% CI Lower–upper	Female (*n* = 80)	95% CI Lower-upper
Age (years)*	56.67 ± 13.12	51.77 – 61.57	51.81 ± 10.39	49.50 – 54.12
BMI (kg/m^2^)	24.71 ± 2.93	23.6 – 25.80	24.32 ± 3.61	23.52 – 25.12
WC (cm)***	96.83 ± 5.77	94.68 – 98.99	92.98 ± 6.72	91.48 – 94.47
WHR***	1.01 ± 0.003	0.99 – 1.02	0.97 ± 0.004	0.95 – 0.97
TC (mmol/L) ***	2.42 ± 0.41	2.27 – 2.58	2.23 ± 0.26	2.17 – 2.29
TG (mmol/L) ***	1.82 ± 0.37	1.68 – 1.96	1.65 ± 0.24	1.60 – 1.71
HDLc (mmol/L)	1.06 ± 0.13	1.01 – 1.12	1.10 ± 0.11	1.08 – 1.13
LDLc (mmol/L) ***	3.64 ± 0.88	3.30 – 3.97	3.23 ± 0.59	3.09 – 3.36
VLDLc (mmol/L) ***	0.36 ± 0.007	0.33 – 0.39	0.33 ± 0.004	0.32 – 0.34
TC:HDLc***	2.32 ± 0.566	2.11 – 2.54	2.04 ± 0.39	1.96 – 2.13
FBG (mmol/L) ***	6.46 ± 2.14	5.67 – 7.26	5.22 ± 1.17	4.96 – 5.48
SBP (mmHg)	145.57 ± 23.41	136.83 – 154.31	149.19 ± 21.69	144.36 – 154.01
DBP (mmHg)	88.43 ± 9.13	85.02 – 91.84	87.71 ± 9.02	85.71 – 89.72

CI =Confidence interval; values are mean ± standard deviation. BMI = Body mass index; WC = waist circumference; WHR = waist–hip ratio; TC = total cholesterol; TG = triglyceride; HDLc = high density lipoprotein; LDLc = low density lipoprotein; VLDLc = very low density lipoprotein; FBG = fasting plasma glucose; SBP = systolic blood pressure; DBP = diastolic blood pressure. Significant sex difference at

* P < 0.05;

** P < 0.01;

*** P < 0.001.

However, males without MS had significantly higher mean WC (*P* < 0.05); WHR (*P* < 0.001); TG (*P* < 0.05); VLDLc (*P* < 0.05), and FBG (*P* < 0.01) as compared to females without MS [Table 2].

**Table 2:** Obesity measures, metabolic profiles, and blood pressures variables between males and females without metabolic syndrome (n = 240)

Variables	Male (*n* = 154)	95% CI Lower–upper	Female (*n* = 86)	95% CI Lower-upper
Age (years)***	53.53 ± 12.24	51.58 – 55.47	45.38 ± 11.80	42.85 – 47.91
BMI (kg/m2)	21.91 ± 4.13	21.25 – 22.56	22.15 ± 4.75	21.13 – 23.17
WC (cm)*	88.44 ± 10.13	86.83 – 90.05	85.12 ± 10.50	82.86 – 87.37
WHR***	0.96 ± 0.006	0.95 – 0.97	0.92 ± 0.005	0.91 – 0.93
TC (mmol/L)	2.28 ± 0.28	2.23 – 2.32	2.24 ± 0.24	2.19 – 2.30
TG (mmol/L)*	1.57 ± 0.27	1.53 – 1.61	1.49 ± 0.23	1.44 – 1.54
HDLc (mmol/L)	1.15 ± 0.12	1.13 – 1.17	1.16 ± 0.12	1.14 – 1.19
LDLc (mmol/L)	3.34 ± 0.65	3.23 – 3.44	3.28 ± 0.58	3.16 – 3.41
VLDLc (mmol/L)*	0.31 ± 0.005	0.30 – 0.32	0.29 ± 0.004	0.28 – 0.30
TC:HDLc	2.02 ± 0.44	1.95 – 2.09	1.96 ± 0.37	1.87 – 2.04
FBG (mmol/L) **	4.92 ± 0.88	4.78 – 5.06	4.64 ± 0.51	4.53 – 5.75
SBP (mmHg)	130.52 ± 23.43	126.79 – 134.25	126.07 ± 21.65	121.43 – 130.71
DBP (mmHg)	81.01 ± 11.44	79.19 – 82.83	79.55 ± 10.39	77.32 – 81.77

Values are mean ± standard deviation. Significant sex difference at

* P < 0.05;

** P < 0.01;

*** P < 0.001.

Comparison of dietary intake of fatty acids (g/week) between males and females with and is presented in Table 3. It was observed that males with MS had a significantly higher consumption of MUFA (*P* < 0.01) and TFA:SFA (*P* < 0.001), whereas females with MS had significantly higher rate of proportion of SFA:MUFA (*P* < 0.01) and SFA:PUFA (*P* < 0.001) consumption than their male counterparts.

**Table 3:** Dietary fat intake (g/week) by males and females with metabolic syndrome (n = 110)

Variables	Male (*n* =30)	95% CI Lower–upper	Female (*n* = 80)	95% CI Lower-upper
TF	615.78 ± 75.46	587.6 – 643.9	586.23 ± 74.53	569.6 – 602.8
SFA	190.43 ± 38.58	176.0 – 204.8	195.82 ± 27.68	189.6 – 201.9
MUFA**	228.60 ± 27.00	218.5 – 238.6	213.72 ± 25.65	208.0 – 219.4
PUFA	159.09 ± 25.76	149.4 – 168.7	153.82 ± 26.79	125.6 – 182.0
TF:SFA***	3.30 ± 0.35	3.13 – 3.47	3.01 ± 0.26	2.9 – 3.0
SFA:MUFA***	0.83 ± 0.11	0.78 – 0.87	0.91 ± 0.10	0.89 – 0.94
SFA:PUFA***	1.21 ± 0.25	1.12 – 1.31	1.41 ± 0.26	1.35 – 1.47
PUFA:MUFA	0.69 ± 0.009	0.66 – 0.73	0.72 ± 0.006	0.58 – 0.86

Values are mean ± standard deviation. TF = Total fat; SFA = saturated fatty acids; MUFA = monounsaturated fatty acids; PUFA = polyunsaturated fatty acids. Significant sex difference at *P < 0.05;

* P < 0.05;

** P < 0.001.

*** P < 0.001.

However, males without MS had significantly higher proportion of TF:SFA (*P* < 0.001) consumption than their female counterparts. Whereas females without MS had significantly higher SFA (*P* < 0.005) and SFA:MUFA (*P* < 0.001) consumption as compared to males without MS [Table 4].

**Table 4:** Dietary fat intake (g/week) by males and females without metabolic syndrome (n=240)

Variables	Male (*n* = 30)	95% CI Lower–upper	Female (*n* = 80)	95% CI Lower-upper
TF	560.09 ± 67.29	549.3 – 570.8	561.30 ± 71.36	546.0 – 576.6
SFA*	146.06 ± 20.18	142.8 – 149.2	152.47 ± 22.89	147.5 – 157.3
MUFA	215.33 ± 18.11	212.4 – 218.2	213.32 ± 19.47	209.1 – 217.5
PUFA	157.26 ± 34.59	151.7 – 162.7	153.47 ± 32.23	146.5 – 160.3
TF:SFA***	3.85 ± 0.28	3.80 – 3.89	3.70 ± 0.26	3.6 – 3.7
SFA:MUFA***	0.67 ± 0.006	0.66 – 0.68	0.71 ± 0.007	0.69 – 0.73
SFA:PUFA	1.00 ± 0.75	0.88 – 1.12	1.02 ± 0.20	0.97 – 1.06	
PUFA:MUFA	0.72 ± 0.12	0.70 – 0.74	0.71 ± 0.11	0.69 – 0.47	

Values are mean ± standard deviation. Significant sex difference at

* P < 0.05;

** P < 0.01;

*** P < 0.001.

Pearson’s partial correlations (controlling for age and sex) between adiposity (WC and BMI) and dietary fatty acids are presented in Table 5. It was observed that WC had significant correlation with SFA (*P* < 0.01); MUFA (*P* < 0.05) as well as TF:SFA (*P* < 0.01); SFA:MUFA (*P* < 0.01), and SFA:PUFA (*P* < 0.01). BMI had significant correlation with SFA (*P* < 0.05) and TFA:SFA (*P* < 0.05). However, no significant association was observed in individuals with MS after controlling for age and sex. On the other, WC and BMI had significant correlation with SFA:MUFA (*P* < 0.01) in individuals without MS.

**Table 5:** Partial correlation (controlling for age and sex) between dietary fatty acids and adiposity measures

Correlation	TFA	SFA	MUFA	PUFA	TF:SFA	SFA:MUFA	SFA:PUFA	PUFA:MUFA
Total population (n = 350)								
WC	−0.92	0.207**	0.133*	−.571	−205**	0.177**	0.116*	−0.088
BMI	0.03	0.117*	0.081	−.056	−.133*	0.096	0.054	−0.076
Individuals with metabolic syndrome (n = 110)								
WC	0.128	0.045	0.124	−.139	0.107	−0.053	−0.012	−0.158
BMI	0.003	−0.080	−0.003	−.103	0.164	−0.110	−0.069	−0.102
Individuals without metabolic syndrome (n = 240)																
WC	−.001	−.036	0.118	−.027	0.060	−0.165**	0.031	−0.108
BMI	−.035	−.082	0.097	−.037	0.082	−0.214**	−0.016	−0.107

Significant at

* P<0.05;

** P<0.01.

## DISCUSSION

Our findings hinted that intake of saturated fat may be a major risk factor for the onset of MS in adult Asian Indians. Moreover, it appears that not the total fat but the amount of saturated fat consumed in association with TFA:SFA, SFA:MUFA, and SFA:PUFA was adversely affecting the adiposity level, lipids, blood pressures, and blood glucose levels in this population and in turn cumulatively enhancing the possibility to predispose to MS phenotypes.

The gender differences in prevalence of MS have been found in several other studies also.[3,19] It might be due to different cut-off points set as criteria for MS like WC and HDLc. It is important to mention that individuals with impaired glucose tolerance, impaired fasting glucose was observed more frequently in men whereas impaired glucose tolerance occurred relatively more often in women.[16,17] Lipids accumulation patterns differ between women and men. Premenopausal women more frequently develop peripheral obesity with subcutaneous fat accumulation whereas men and postmenopausal women are more prone to central or android pattern of obesity. In particular, android obesity is associated with increased cardiovascular mortality and the development of T2DM. Inflammation increases cardiovascular risk particularly in women. It has also been mentioned that the pathophysiology of the MS, and its contribution to the relative risk of cardiovascular events and heart failure show gender differences, which has immense potential relevance for prevention, diagnostics, and therapy of the syndrome.[20]

Certain dietary patterns, including high SFA and low vegetable intake, contribute to weight gain and increase the risk of metabolic disturbances, whereas such potentially modifiable lifestyle factors may reduce cardiovascular risk.[10,21] In a recent study from India revealed that increased dietary ω-6 PUFA and saturated fat intake are significantly associated with fasting hyperinsulinemia and subclinical inflammation, respectively, and might be responsible for the increasing prevalence of insulin resistance, the MS and T2DM in Asian Indians.[8,9,16,22] In another study, it was found that the dietary total fat may increase whereas linoleic acid intake may reduce the risk of MS in Japanese descendants living in Brazil.[23] Study from the United Arab Emirates (UAE) pointed out that poor dietary habits including consumption of high-energy foodstuffs, diets high in total carbohydrates, fat, and simple sugars were associated with MS.[24] In a multiethnic study (comprising of African-Americans, Whites and Hispanics) on healthy children aged 7–12 years revealed that diet composition was more closely related to the components of the MS than was physical activity, with carbohydrate intake being adversely related to WC, TG levels, and glucose levels. Moreover, relationships among diet and MS outcomes were stronger among African-American children[25] reflecting ethnic variation. Our findings of an adverse impact of TFA:SFA intake and inverse relation of adiposity with PUFA, i.e., protection of polyunsaturated fat concerning the association with MS is in accordance with the finding of several other investigations.[23,26–29]

However, the major limitation was that the study was performed on a relatively small sample size and therefore not representative of Asian Indian population. Moreover, the dietary fatty acids were obtained through retrospective method and not directly from the isolated plasma. Further prospective studies are required to better compared *gene-diet* interaction 30 in the growing menace of MS in this part of the world.
